# Bereavement practices within older adult care homes in Scotland: a focus group study

**DOI:** 10.1136/bmjopen-2025-115592

**Published:** 2026-02-23

**Authors:** Maria Drummond, Jennifer Burton, Doreen MacEachen, Bridget Margaret Johnston

**Affiliations:** 1 Nursing and Healthcare, School of Medicine, Dentistry & Nursing, University of Glasgow College of Medical Veterinary and Life Sciences, Glasgow, UK; 2 ENRICH Scotland, NHS Research Scotland, Scotland, UK; 3 Academic Geriatric Medicine, School of Cardiovascular and Metabolic Health, University of Glasgow College of Medical Veterinary and Life Sciences, Glasgow, UK; 4 NHS Greater Glasgow and Clyde, Glasgow, UK

**Keywords:** PALLIATIVE CARE, Nursing Homes, Nursing research

## Abstract

**Abstract:**

**Objective:**

To describe how care home staff experience bereavement and their perspectives on providing bereavement care within care home settings.

**Design:**

Qualitative descriptive study using focus groups analysed with the Framework Method.

**Setting:**

Seven residential and nursing care homes for older adults in Scotland.

**Participants:**

37 care home staff were recruited through the Enabling Research In Care Homes (ENRICH) Scotland research network. Participants included registered nurses, care workers, senior care workers, managers and ancillary workers with experience of resident death and bereavement practice.

**Results:**

Bereavement was woven through everyday care home life, understood as a tapestry of experiences, relationships and practices that involved staff, residents and their relatives. Three themes that connected to the tapestry metaphor were identified: Warps: structural threads grounding bereavement within the culture of homely living, where close bonds normalise death and dying, and pragmatic acceptance. Wefts: strengthening practices nurturing resilience, including relational trust, mutual support, rituals and follow-up with relatives. Moths: disruptions undermining bereavement practice include family secrecy, hospital deaths with withheld information, difficulty supporting residents with advanced dementia and dissatisfaction with online training.

**Conclusions:**

Bereavement in care homes is collective, relational and embedded in routine practice. Organisational recognition of grief improved interservice communication. Tailored reflective support for staff is needed to sustain compassionate care. Further research should explore how residents experience repeated exposure to death and bereavement within communal living environments.

**STRENGTHS AND LIMITATIONS OF THIS STUDY**

- Inclusion of staff across diverse roles and seniority levels enabled capture of organisationally embedded bereavement experiences beyond clinical perspectives alone.
- On-site focus groups within participating care homes supported context-rich discussion grounded in everyday practice, enhancing ecological validity.
- Use of fictional scenarios provided a consistent stimulus across groups, facilitating comparison while enabling participants to reflect safely on sensitive experiences.
- Voluntary participation and recruitment through a research-engaged network may have introduced selection bias towards homes already motivated to reflect on bereavement care.
- This study only recruited care home staff, and because of this, the views and opinions of care home residents and their relatives are not represented in the data and interpretation.

## Introduction

 Globally, care homes have become central settings for end-of-life care, with a significant proportion of older adults spending their final months or years here.^1 2^ In Western countries, care homes account for a growing share of deaths among older populations, with estimates of 21–37% of all deaths occurring in care homes.^3 4^ This figure is projected to increase substantially as the proportion of older people increases.^5^ Care home staff are, therefore, not only essential providers of care at the end of life, but also frequent witnesses to death and grief.^6^

In Scotland, average life expectancy among older adults living in care homes is around 2 years.^7^ However, the individual timing of mortality is highly variable, with some residents dying the day they move in to the care home and others living many years there.^8^ Thus, care home staff face a dynamic situation around their relationship with and knowledge of the individual resident at the end of their life.^8^ Care home staffs’ involvement in the postdeath period of resident care extends beyond care responsibilities to the resident to include emotional support for other residents, families of the deceased and colleagues.^9 10^ This repeated and intimate exposure to dying and bereavement places staff in a complex position. They support others through grief while experiencing their own emotional responses to the deaths of residents with whom they may have formed close bonds.^11 12^ Care assistants have been identified as a key group to prepare, train and support in international evidence.^13^ The emotional labour associated with consistent exposure in work settings to death and dying can have significant emotional, physical and spiritual ramifications that require workplace mitigation strategies.^14^,^16^

Despite this, research on bereavement practice within UK care homes is limited and practice is inconsistently evaluated.^17^ There is a particular lack of evidence on how care home staff support other residents following a resident’s death.^18 19^ The organisational context operating within care homes is recognised to influence how staff experience grief and loss.^20^ Given the growing international recognition of bereavement as a public health concern, understanding and supporting care home staff is essential for workforce well-being and sustainable, compassionate care delivery.^21 22^ Therefore, this study describes the experiences and perspectives of care home staff on bereavement through focus groups conducted in Scottish care homes. It aims to deepen understanding of the emotional, relational and organisational dimensions of grief in this setting. It is reported in line with the COREQ (Consolidated criteria for Reporting Qualitative research) checklist^23^ (see online supplemental appendix 1).

## Methods

### Aim

To describe how care home staff experience bereavement and their perspectives on providing and delivering bereavement care to others within care home settings.

### Study setting

In Scotland, care homes for older adults provide 24-hour support for people who can no longer live independently. These settings offer accommodation, personal care (such as help with washing, dressing and mobility) and nursing care either from on-site nurses (nursing homes) or National Health Service (NHS) community nursing services (residential homes). Care home residents are a frail population who account for a significant proportion of deaths in older age groups in Scotland, reflecting the routine presence of dying and death within care home settings.^7 8 24^ In Scotland, national policy frames palliative and end-of-life care as a priority centred on dignity, person-centred care and family support.^25^ However, variation in practice and integration with wider services remains a continuing challenge. UK evidence highlights persistent difficulties in coordination between care homes and external services.^26^ This disconnect becomes particularly visible when residents deteriorate. In these situations, care home staff regularly weigh complex risks and benefits under conditions of uncertainty, shaped by relationships and organisational pressures.^27^ Nevertheless, most care homes report that they have the capacity to support and deliver palliative care. Care homes are regulated by the Care Inspectorate and may be run by local authorities, private providers or voluntary/not-for-profit organisations.

### Study design

This study was situated within the interpretivist paradigm, which recognises that knowledge is shaped by context, experience and interpretation.^28^ Given the aim of describing how care home staff experience and provide bereavement support, a qualitative descriptive methodology^29^ was employed to capture participants’ perspectives in their own words, with minimal abstraction. This approach is well-suited to research that seeks to inform practice and policy.^30^ Data were analysed using the Framework Method,^31^ which provides a structured but flexible approach to managing and interpreting rich qualitative data analysed by interdisciplinary teams.

The study was conducted in Scotland in partnership with Enabling Research In Care Homes (ENRICH) Scotland.^32^ ENRICH Scotland is funded by the chief scientist office to support research engagement and capacity-building in care homes. This research was developed to explore care home perspectives as part of a wider project developing a Bereavement Support Toolkit.

### Recruitment and participants

Care homes were recruited through the ENRICH Scotland network of Research Ready care homes.^32^ Managers of Research Ready homes were contacted by email by a member of the ENRICH Scotland team, independent from the research team. Once a manager expressed interest, they were sent an electronic participant information sheet and consent form. Individual written consent was then confirmed and obtained in person from all participants at the beginning of each focus group.

Inclusion criteria**:** Eligible sites were care homes in Scotland registered with the Care Inspectorate that provided 24-hour care for older adults. Eligible participants were staff aged ≥17 years, employed in participating homes in any role with experience of resident death or bereavement practice and able to provide informed consent.

### Research team

The focus groups were led by MD (female, PhD), a postdoctoral nurse researcher with clinical and research experience in care homes. Participants were made aware of MDs’ experience as a researcher and community nurse prior to starting the meetings and purpose of the focus groups. Data collection was supported by a member of the ENRICH Scotland team (one male and two females) or our research assistant (DM, female), all of whom are registered nurses with experience in older adult care. The cofacilitators assisted with logistics and helped ensure a supportive group environment, including being available to provide emotional support to participants. They also completed field notes made up primarily of non-verbal observations during the groups. Data analysis was conducted collaboratively by the core research team (MD, JKB, DM, BJ), whose combined expertise spans qualitative methods, ageing and palliative care.

### Patient and public involvement

A patient and public involvement (PPI) representative (a former care home relative and carer) became involved early on in the project during protocol development. She contributed to the development of discussion materials used during the focus groups and reviewed the developing analytical framework and themes.

### Data collection

Recruitment was open between August and December 2024, with seven focus groups conducted between October and November of that period. All groups took place on-site at participating care homes, in quiet and private rooms chosen to promote open discussion. Descriptive data were collected about each care home service and basic demographics were collected from participants.

To prompt discussion, participants were presented with four fictional scenarios, each based loosely on common care encounters drawn from the project lead’s experience. These were developed collaboratively with the research team and PPI representative (see online supplemental appendix 2). Participants were invited to reflect on how they would care for the individuals described in each scenario. Focus groups were audio-recorded and transcribed verbatim by an independent professional transcription service. Transcripts were analysed using Microsoft Word (Office V.16) and stored on a secure University drive.

### Data analysis

The Framework Method involved familiarisation with the data, developing a thematic framework after reviewing the first two transcripts, indexing transcripts, charting data into a matrix and interpreting patterns across cases and themes. Initial coding and matrix charting were conducted by the project lead (MD) and two team members (DM and JB), with regular full team discussions to ensure consistency and rigour.

### Rigour

In qualitative research, the concept of trustworthiness is appropriate for evaluating rigour. Trustworthiness encompasses several dimensions, which may include credibility, dependability, confirmability, transferability and authenticity.^33^ In this study, we have enhanced credibility through transparent reporting of data collection and analysis methods, and authenticity by detailing our recruitment processes and participant context. To support transferability, the findings have been situated within the existing literature. For analytical transparency and to provide an explicit audit trail, a summary thematic table (see table 1) was developed linking codes to overarching themes with illustrative quotes.

**Table 1:** The analytical structure of the thematic framework with corresponding codes and quotes

Theme	Key analytical codes	Illustrative quote
Warps: structural threads	Normalisation of death; acceptance of dying; experiential learning; honesty in communication to support preparation	“P3, RN: You have to be honest, and because that also prepares them as well, because if you use a term that sort of like goes in circles, you are not allowing them to be in the same page with you, because then they’ll be thinking, oh the person’s going to be here forever. P1, CW: And they’re going to get better. P3, RN: And they’re going to get better, they’re going home and things like that and it’s not going to be like that. So, it’s better to do the Death and Dying as death and dying and then, yeah, that way everyone will be prepared, the staff and the resident.” - FG 6
	Rapid emotional switching; self-preservation; professional masking; continuity of care	“P6, RN:(You’re with)Someone dying to then maybe go and do someone’s dressing, maybe do someone’s catheter or then you maybe go in the corridor, and you see another family, they’re… P4, RN: Self-preservation… You have to go that situation, just smiling and being nice to someone. P6, RN: Yeah, you need to have a positive interaction with them where you’ve just come…but then they don’t know what you’ve just dealt with so… P1, RN: You have to learn how to put things in boxes.” - FG7
	Deep relational bonds; shared grief; collective belonging; home versus workplace	“We’ve got quite a few residents who the family will say that we’re more family now than them because they’re, kind of…their visits are fleeting while we’re here all the time” - FG3, P2, SCW.
Wefts: strengthening threads of care and connection	Long-term relationships; familiarity; shared life history; continuity	“We build up a great rapport with family members… we’ve got that bond already to offer support. We’ve already established that connection, which is really, really important” - FG1, P2, activity coordinator.
	Peer support; humour; shared coping; collective resilience	“I think supporting each other is a big thing. ‘Cause I think, like, if we didn’t have support just, say, from the nurses, I think I would feel differently. I would be…you would question yourself more. But ‘cause we’ve got the back up of our nurses and, like, we back them up, I think it makes a big difference, supporting each other.” - FG7, P5, CW with audible group agreement.
	Memorial acts; marking death; storytelling; symbolic continuity	Online supplemental appendix 4
Moths: disruptions and fraying of bereavement practice	Communication barriers; protection versus exclusion; moral distress	“The most recent gentleman that passed away, his family hadn’t told his wife that he was dying. So that conversation couldn’t have been had. So, they hadn’t told her that he was palliative care ‘cause the daughters were not wanting her to know, they were wanting her to enjoy every minute with him, without knowing that he was actually dying.” - FG3, P4, SCW.
	Disconnection from dying process; lack of closure; frustration with services	“P3 CW: And if they’re do die here then we get to know what’s on the death certificate, but if they die in hospital, we don’t, unless the family lets us know. P4 RN: Maybe the GP will let you know if you ask them. P2 RN: But it’s up to the hospital if they die in hospital, so…we don’t usually get to find out.” - FG7
	Invisible grief; cognitive impairment; ethical tension	“Some care homes say, they came through the front door and will leave through the front door. We don’t do that. We try to distract the residents. It’s not that we don’t want to show the respect to this person, but that will affect probably all other 39. They will not know what’s happening and they will be confused where they are if they think this is a hotel, so why do you see a coffin in a hotel? A lot of things, we need to do things differently in dementia service.” - FG5, P3, CW
	Tick-box education; lack of emotional preparation; skills gap	“Because it’s hard, these days you do everything online. We need it face to face and that was great. You can’t feel staff emotions when you do online training, and that’s very emotional training” - FG5, P3, CW.

CW, care worker; RN, registered nurse; SCW, senior care worker.

## Results

### Participating care homes and care home staff characteristics

20 care home managers expressed interest and were sent participant information to review. Nine focus groups were arranged; however, two managers dropped out due to staffing (n=1) and then concerns about their suitability (n=1). Of the seven focus groups that were completed, 37 people participated and included registered nurses, care workers, senior care workers, domestic assistants, activities coordinators, receptionists and managers (see online supplemental appendix 3 for more details).

The participating care homes varied in size, from 27 to 80 places (average 44 places). Four were private providers and three voluntary/not-for-profit providers with no local authority provided services recruited. Four homes were situated in urban areas and three in rural ones. All homes provided 24-hour care for older adults, including specialist care for people living with dementia (see online supplemental appendix 3 for more details).

### Thematic overview

From the focus group data, the research team came to understand bereavement as part of a wider tapestry of care woven from the care home itself. Staff weave threads that include their own experiences and relationships and that each resident, relative and visitor can contribute to. These become interlaced to create a shared fabric of community life. This collective tapestry reflects that life, death, grief, remembrance and support are interconnected in the care home setting.

My mum died when there was a lady in this room here dying and they both died within an hour of each other, but I was on shift, so I’d had a phone call, but I was with this other family… - FG7, P4, registered nurse

Within the tapestry, we identified key components of bereavement practice from staff focus group data which formed three themes of:

- Warps: structural threads creating the integrity of bereavement and grief.
- Wefts: strengthening threads of care and connection.
- Moths: disruptions, gaps and missed connections that threaten to fray or unravel what has been created.

This metaphor acknowledges the richness and fragility of bereavement practices in care homes. Every thread matters, and the strength of the fabric depends on the care invested in weaving it and the attention paid to repairing what is worn or at risk.

Table 1 provides an overview of this thematic framework, outlining key analytical codes and illustrative quotes onto the three overarching themes to support interpretation of the findings that follow

### Theme one: the warps

When participants described bereavement care, the research team identified the primary warp thread as death existing as a cultural constant within a truly homely setting. This grounds care homes as communities where death is normalised and where bonds between staff, residents and families can create enduring connections.

Nursing and care staff across the focus groups described instances where residents had expressed an acceptance and readiness of death. Rather than recounting useful training on advanced communication or palliative care, their confidence in navigating and sharing these conversations came from *‘*experience. Just experience’ (RN, FG7). Indeed, this regularity and experience highlighted the importance of using ‘straightforward’ language to help all those involved prepare for the inevitable:

P3, RN: You have to be honest, and because that also prepares them as well, because if you use a term that sort of like goes in circles, you are not allowing them to be in the same page with you, because then they’ll be thinking, oh the person’s going to be here forever.

P1, CW: And they’re going to get better.

P3, RN: And they’re going to get better, they’re going home and things like that and it’s not going to be like that. So, it’s better to do the Death and Dying as death and dying and then, yeah, that way everyone will be prepared, the staff and the resident. - FG 6

Care and nursing staff described how they must shift swiftly from the emotional weight of supporting end-of-life and bereavement to the routine of everyday care for well residents and interactions with families and relatives who may be unaware of the loss that has just occurred:

P6, RN: [You’re with] Someone dying to then maybe go and do someone’s dressing, maybe do someone’s catheter or then you maybe go in the corridor, and you see another family, they’re…

P4, RN: Self-preservation… You have to go that situation, just smiling and being nice to someone.

P6, RN: Yeah, you need to have a positive interaction with them where you’ve just come…but then they don’t know what you’ve just dealt with so…

P1, RN: You have to learn how to put things in boxes. - FG7

Many participants felt that acknowledging death openly, including with residents, respected the integrity of the community but that the timing and approach of how it is done required nuance and care:

You know, it’s the residents’ right to know their neighbour or their friends has passed away. - FG1, P7, manager

Indeed, many participants described the care home as more than a workplace or service; it was home, where relationships with residents and their relatives accepted, understood or identified that family-like attachments can form:

We’ve got quite a few residents who the family will say that we’re more family now than them because they’re, kind of…their visits are fleeting while we’re here all the time. - FG3, P2, SCW

Across the focus groups, breaking bad news to relatives was tasked to senior staff. They described how they played a central role in orchestrating farewells and the importance of maintaining honesty with families. However, ancillary staff, such as administrators and domestic assistants, described how they would make themselves available for relatives who they knew well. The tone and timing of communication, including non-verbal, tactile communication, were described as central to supporting relatives through bereavement, with staff drawing on the trust built during ongoing relationships:

And, you know, this lady would come in, and [Administrators Name] would be probably the first one she would speak to… And the first person to offer a cuddle, you know, that’s typically how it would probably work here. - FG2, P1, manager

The repeated exposure to death and grief experienced by all staff groups fostered a confident and pragmatic acceptance. While these experiences could be emotionally demanding, they also shaped a shared foundational understanding of mortality as part of life in the care home.

### Theme two: the wefts

Woven across the structural threads were the wefts: strengthening practices that nurtured resilience, reinforced bonds and ensured bereavement was met with compassion. Senior carers, nursing and management staff frequently emphasised the importance of planning for end-of-life and bereavement, while also recognising the sensitivities involved. They described how, ideally, care plans would be developed with relatives. Good care plans were described as ‘living’ documents that capture specific wishes, for example, preferred place of care or funeral arrangements, and are revisited as needs and wishes change. Where possible, residents themselves would also be involved, but rather than relying on direct approaches, staff also paid attention to subtle expressions of readiness to probe further and document later:

if… they are saying things like, you know, och, I’m done in, I’m done in… that’s usually, like, an open question to be like, right, okay, well what do you mean by that. And they just say, I don’t want to do this, I don’t want to do that, and you can you just have a, kind of, insight into what they’re wanting. - FG3, P7, RN

Our PPI member reinforced the importance of thoughtful care plans as strengthening threads. She reflected on how advance discussions gave her family the confidence to support her mother in the care home rather than transferring her to hospital, and how Namaste Care in her mother’s final days provided comfort and dignity for both mother and daughter.

Alongside formal planning, relational bonds underpinned how bereavement care was experienced. Time spent building rapport with families meant that when death occurred, staff in a variety of roles were already positioned as trusted supporters:

We build up a great rapport with family members… we’ve got that bond already to offer support. We’ve already established that connection, which is really, really important. - FG1, P2, activity coordinator.

The importance of ensuring that nobody died alone was a consistent thread across focus groups. Staff felt strongly that the presence of carers or colleagues at the bedside reflected the dignity of the individual, even in the absence of family:

But we’ve also had residents who have not had any family, and they’ve had staff that have… been right beside them. - FG3, P5, manager.

Staff also described how bereavement was carried collectively, with mutual support among colleagues helping them to manage their own grief. These interactions were often informal, based on what they knew about each other and their relationships with the residents involved, but deeply meaningful. Participants from all staff groups described moments of shared reflection during handovers or breaks, or managers with open door policies, providing opportunities for care and reassurance:

I think supporting each other is a big thing. ‘Cause I think, like, if we didn’t have support just, say, from the nurses, I think I would feel differently. I would be…you would question yourself more. But ‘cause we’ve got the back up of our nurses and, like, we back them up, I think it makes a big difference, supporting each other. - FG7, P5, CW with audible group agreement.

These social rules would entwine around more structured rituals, practices and memorialisation opportunities described by participants (online supplemental appendix 4). Collectively, they created wefts that strengthened the tapestry through relational capital, compassionate rituals and an understanding that emotional labour was shared. Managers and registered nurses who accommodated more structured support and activities into everyday practice ensured that bereavement was carried together.

### Theme three: the moths

Despite the strength of these threads, staff described vulnerabilities in bereavement practices that risked fraying the tapestry. These ‘moths’ represented gaps and challenges that weakened the ability of staff to feel confident that they were providing care that aligned with residents’ preferences and risked disrupting their own grief.

Family secrecy and denial were among the most difficult disruptions described by nursing and care staff. They recalled situations where relatives withheld information from one another, making open conversations about death and future care planning (FCP) seem impossible:

The most recent gentleman that passed away, his family hadn’t told his wife that he was dying. So that conversation couldn’t have been had. So, they hadn’t told her that he was palliative care ‘cause the daughters were not wanting her to know, they were wanting her to enjoy every minute with him, without knowing that he was actually dying. - FG3, P4, SCW.

Participants also pointed to the limits of current training provision trends. While many staff were eager for opportunities to reflect and learn together, online modules were felt to be inadequate for such complex work:

Because it’s hard, these days you do everything online. We need it face to face and that was great. You can’t feel staff emotions when you do online training, and that’s very emotional training. - FG5, P3, CW.

Staff bereavement was made more complex when residents died in hospital. A nurse used the group discussion to reflect further on clinical decision-making that led to a hospital admission for a much-loved resident, which had occurred months prior. Even though they described feeling ultimately ‘comfortable’ with the hospital admission, their body language, tone of voice and the reassurance given in the room suggested there was a knot that persisted in their grief:

So, you make that decision, you call the doctor, and you call the ambulance, so I’m comfortable, ‘cause I have reflected on it obviously, but I am comfortable with the decisions I made on that day. But it’s just, like, it, kind of, stopped with the doctor. At the end of the day, I’m presenting to you what I think. You’re the one that’s…you’re above me. You say you’re coming/you’re not coming. But then maybe I could have pushed a bit more, but then sometimes how can…what can…how much can you challenge someone… Yeh I didn’t expect him to pass away. - FG7, P6, RN

Nursing and senior care staff were also sometimes excluded by hospital staff from communication about residents’ death, even when they had cared for residents for many years. Other than the practical challenges this creates for care home staff trying to fulfil regulatory reporting requirements, this lack of closure seemed to intensify grief and create unanswered questions, particularly for those involved in the decisions that led to the admission:

P3 CW: And if they do die here then we get to know what’s on the death certificate, but if they die in hospital, we don’t, unless the family lets us know.

P4 RN: Maybe the GP will let you know if you ask them.

P2 RN: But it’s up to the hospital if they die in hospital, so…we don’t usually get to find out. - FG7

Supporting bereaved residents with cognitive impairment posed further dilemmas. Some staff believed residents, including those with advanced dementia, still sensed absence and deserved open communication:

I think it’s always, like, fallacy to think that residents don’t know when somebody’s passed, because they do. And I think even the people that are quite advanced with their dementia still recognise that there’s a void, like that chair’s suddenly empty when it wasn’t before. - FG3, P1, CW

Whereas others worried that such disclosure and formal recognition could cause confusion or distress, especially when residents with more advanced cognitive impairment perceived the home as a temporary setting:

Some care homes say, they came through the front door and will leave through the front door. We don’t do that. We try to distract the residents. It’s not that we don’t want to show the respect to this person, but that will affect probably all other 39. They will not know what’s happening and they will be confused where they are if they think this is a hotel, so why do you see a coffin in a hotel? A lot of things, we need to do things differently in dementia service. - FG5, P3, CW

These moths reflect the varied challenges faced by care home professionals and the need for bereavement practice to be adapted by care homes to meet the needs of the diverse population living, dying and working there rather than a fixed set of components for all homes to evidence.

### Reflexivity

The study was originally designed to recruit staff working in direct care roles. However, domestic assistants, administrative staff and activity coordinators also participated. Braun and Clarke^34^ advise that researchers should be prepared for the unexpected on data collection days. Indeed, participants from these roles gave valuable insights that helped create a more detailed understanding of how a care home forms its own tapestry of care and connection.

The lead researcher’s (MD) professional background provided familiarity with the context, language and organisational dynamics of care home and end-of-life practice, which supported rapport building during focus groups. At the same time, her close familiarity with the setting required ongoing reflexive attention to ensure that participants’ experiences were not interpreted through an overly clinical lens or through her own experiences. Regular team discussions and post focus-group debriefs between the facilitators were used to surface and reflect on assumptions.

## Discussion

This study explored bereavement care in Scottish care homes through the metaphor of a tapestry: staff weave threads from their relationships, experiences and memories into a fabric made from care home life, which intrinsically includes death and grief. The findings reveal that care home life is ingrained with death and grief. In contrast to literature that has largely focused on advance care planning or clinical processes,^9 35^ this study conceptualises bereavement as a collective, everyday practice embedded within care home communities. Staff (including ancillary staff) are deeply emotionally and professionally invested in providing meaningful and intuitive bereavement support. Key practices relate to the integration of bereavement as an extension of end-of-life care. Care home staff encourage informal debriefing alongside openness about death, memorial activities, funeral attendance and personalised support for those grieving. Recent research, which also included family caregivers,^36^ has similarly demonstrated that grief for families begins when a resident moves in and unfolds across the trajectory, further highlighting the importance of rituals and community. However, the grief that staff experience is interlaced with the death experienced by the resident. Therefore, when death occurs in hospital, is due to unexpected circumstances or when residents’ preferences are unknown, staff can experience emotional distress linked to uncertainty about their care decisions. In homes supporting people with advanced dementia, staff described many standard bereavement practices and rituals as unsuitable. These findings have practical implications for how bereavement is recognised, supported and resourced within care homes, particularly in relation to workforce support, communication with external services and dementia-specific guidance.

Decision-making around the care of residents can be fraught with uncertainty when acute health changes necessitate decisions about hospital admission, especially when the resident is approaching the end of their life.^37^,^39^ Focus group participants highlighted the emotional and ethical complexity of such decisions when residents die during their hospital stay. We heard that staff are sometimes left without clear information about the cause or circumstances of death, which can intensify feelings of helplessness and prolong their grieving process. Such moments underscore the dual burden care home staff carry, of making time-pressured decisions with incomplete prognostic certainty while anticipating the personal and professional aftermath of those decisions.^27^ These findings add to what is known about the hospital-to-care-home disconnection. This adds urgency to the need for improved communication pathways between hospitals and care homes, as well as structured support systems for staff who routinely navigate these emotionally complex scenarios.^38 40^

This study identified wider tensions in practice about how and when to communicate the death of a resident to other residents, particularly to residents with cognitive impairment and dementia. This is despite evidence that people with dementia can retain capacity to know that the death of a loved one has occurred and may experience grief even in later stages of the condition.^41^ Moreover, they might also not necessarily be significantly or overly negatively impacted by the news.^18^ Avoiding conversations about death risks limiting residents from mourning and may lead to expressions of grief being misinterpreted as behavioural symptoms.^42^ Supporting residents to grieve requires careful, compassionate communication and a willingness to recognise the social connectedness that can exist between residents.^42 43^ Staff in this study used their knowledge of individual residents to make nuanced judgements but highlighted the need for more guidance and confidence in this area. This adds nuance to current dementia bereavement literature by showing how staff rely on relational judgement in the absence of formal guidance. This knowledge gap extends to how care home staff deal with mass bereavement: care homes are especially vulnerable to multiple deaths occurring in a short period due to infectious outbreaks or the inherent frailty of the resident population.^12^ Coordinated, systems-level approaches are required to manage these events, yet few frameworks address this explicitly.^44 45^ Bereavement in care homes must, therefore, be understood as a collective event shaped by relationships and rhythms of communal life.

Grief is intrinsic to the care home environment, woven into the everyday experiences of staff. Care workers and registered nurses routinely witness the decline and death of residents while supporting the everyday care needs of those who remain.^46^ This involves shifting between modes: managing the intensity of death and grief in one moment, then returning to the rhythm of a ‘normal’ day in the next.^47^ This is particularly evident in dementia care; residents are usually unaware of a death, so consistency and calm are essential. The emotional labour of this balancing act can be considerable, highlighting the need for greater recognition and support for those who carry the weight of professional responsibility and human compassion.^48^ This is an aspect of care home work that remains under-recognised in policy and training frameworks.

Participants in this study are not the first to outline challenges associated with FCP when the friends and relatives of residents are reluctant to engage.^49 50^ This may be in part due to cultural attitudes where death is treated as taboo rather than a natural part of life.^51^ While this should not deter engaging in FCP conversations, recognition of the barriers to FCP must be acknowledged in national policymaking/guidance.^52^ Indeed, care home staff are not immune to the cultural discomfort and secrecy that often surround death. This was reflected in expressions of paternalism and uncertainty about when and how to initiate conversations about dying with residents. Such hesitancy stands in contrast to evidence that many care home residents are open and accepting of death, including their own.^18 53^ Our findings suggest this tension may be partly cultural, embedded within everyday care home practice. Involving the wider multidisciplinary team in FCP discussions offers a valuable opportunity to foster positive, open attitudes early.^49^ For care homes, this may include new members of the team and students to help create the confident and compassionate workforce needed to lead these important discussions.^54^

### Strengths and limitations

This study includes a role-diverse sample of care home staff, enabling us to capture a wide range of perspectives on bereavement practice. Participants represented various seniority levels, recruited within their own care home settings, so their reflections arose from real-time experience rather than recollection removed from context. This strengthens the ecological validity of the insights generated and has allowed us to recommend implications for further research.

Conducting the focus groups on-site meant staff were periodically called away to attend to residents; these interruptions shortened some discussions and may have constrained the depth of exploration. However, these situations demonstrated how often care home staff must adapt when working. In addition, participation was voluntary, so the voices heard may disproportionately represent those already motivated to reflect on or improve bereavement care, introducing the possibility of volunteer bias and limiting the transferability of findings to less engaged staff or settings. Finally, this study involved only care home staff as participants. As such, it does not capture the experiences or perspectives of residents or relatives. Given that residents themselves identify communal living in care homes as a research priority,^55^ we suggest that understanding death and bereavement exposure from their perspective is an urgent area for future research.

### Conclusion

Bereavement is a routine yet emotionally significant aspect of care home life, deeply affecting staff across roles. This study highlights the complexity of grief in these settings, shaped by varied resident trajectories, emotionally charged decision-making and organisational cultures. Nevertheless, care home staff navigate these experiences while continuing to provide compassionate care to others. Improving bereavement care in care homes requires systemic recognition of grief as part of everyday practice, investment in staff training tailored to different roles and stronger communication pathways, particularly from hospital staff. Supporting the emotional well-being of staff is essential for workforce sustainability and for ensuring dignified, compassionate care at the end of life.

## Supplementary material

10.1136/bmjopen-2025-115592 online supplemental file 1

10.1136/bmjopen-2025-115592 online supplemental file 2

10.1136/bmjopen-2025-115592 online supplemental file 3

10.1136/bmjopen-2025-115592 online supplemental file 4

## Data Availability

Data are available upon reasonable request.
