# Supplementary material for: Bereavement practices within older adult care homes in Scotland: a focus group study
Source: BMJ Open. 2026 Feb 23;16(2):e115592. doi: 10.1136/bmjopen-2025-115592 (PMC12931547; doi:10.1136/bmjopen-2025-115592)
Supplement: online supplemental file 2 [file bmjopen-16-2-s002.docx]

### Scenario 1: Supporting a Grieving Partner

Background: Margaret and Harold had been married for 55 years. Harold had been a resident in the care home for two years due to his declining health, while Margaret visited him every day, often spending hours by his side. Harold died peacefully but unexpectedly in his sleep one night. He was found by night staff in the morning. Care staff phoned Margaret to ask her to visit the care home urgently. Upon hearing the news, she was visibly devastated and struggled to process the loss, remaining in Harold’s room for several hours, clutching his belongings.

#### Discussion Questions:

- How would you approach Margaret to offer her support in this moment of grief?
- What considerations should be made when informing a long-term partner about the death of a resident?
- How can the care home continue to support Margaret in the days and weeks following Harold’s passing?
- What role does clear communication and empathy play in supporting grieving partners?

### Scenario 2: Comforting an Upset Staff Member

Background: Emily, a care assistant, had developed a close bond with Mr. Thompson, a resident she had been caring for over the past year. They shared daily conversations, and Emily got to know his family well. Mr. Thompson died one morning, and Emily was the one who found him. She was deeply affected, feeling both grief and guilt, wondering if there was more that she could have done.

#### Discussion Questions:

- How should the care home management support Emily in the immediate aftermath of Mr. Thompson’s death?
- What are the best practices for supporting staff members who develop close relationships with residents?
- How can the care team address feelings of guilt and sadness among staff following the death of a resident?
- What ongoing support can be offered to Emily and other staff to help them cope with their emotions?

### Scenario 3: Assisting a Grieving Resident

Background: Betty and Joan had become inseparable friends during their time as residents at the care home. They spent most of their days together, sharing meals, attending activities, and reminiscing about their lives. Joan’s health declined following a chest infection. She received end of life care for around a week and died with family in attendance. The family expressed their gratitude to staff at the care Joan received. However, Betty was inconsolable. She refused to leave her room, skipped meals, and stopped participating in activities. The care team noticed a significant decline in her physical and emotional well-being.

#### Discussion Questions:

- How can the care team support Betty in her grief and encourage her to re-engage with life at the care home?
- What specific interventions could be implemented to help Betty cope with the loss of her friend?
- How can the care home foster a sense of community and support among residents who are receiving palliative and end of life care?
- What role does staff play in monitoring the well-being of grieving residents and offering support?

### Scenario 4: Supporting a Grieving Child of a Deceased Resident

Background: John lives in Australia. His mother, Mrs. Clark, has lived in the care home for 9 months. He was her only child and had taken on the responsibility of managing her care but from a distance. When Mrs. Clark died following a brief illness, John was devastated. He felt an overwhelming sense of loss, coupled with concerns about whether he had made the right decisions regarding her care. He returned to Scotland for her funeral and during this time, contacted the care home regularly to discuss care decisions and the contents of care records.

#### Discussion Questions:

- How should the care team approach John to provide comfort and support following his mother’s death?
- What specific actions can the care home take to help John deal with his grief and any feelings of guilt or doubt?
- How can the care team ensure that John feels supported and informed during the bereavement process, including the logistics following the death?
- What long-term support can be offered to family members like John who were closely involved in the care of their loved ones?
