# Supplementary material for: Bereavement practices within older adult care homes in Scotland: a focus group study
Source: BMJ Open. 2026 Feb 23;16(2):e115592. doi: 10.1136/bmjopen-2025-115592 (PMC12931547; doi:10.1136/bmjopen-2025-115592)
Supplement: online supplemental file 3 [file bmjopen-16-2-s003.docx]

Appendix 3: Characteristics of participating care home services and participants

| **Focus Group** | **SIMD** | **Urban - Rural** | **Bed capacity** | **Care Provided** | **Ownership** | **Characteristics and role of participants** | | | | | | | | | |
| --- | --- | --- | --- | --- | --- | --- | --- | --- | --- | --- | --- | --- | --- | --- | --- |
|  |  |  |  |  |  | **Age Range** | **Sex (Female/Male)** | **AC** | **A** | **CW** | **DA** | **DM** | **M** | **RN** | **SCW** |
| 1 | 5 | 5 | 27 | Residential, Dementia | Voluntary/Not-for-profit | 28-60 | F=4, M=1 | 2 |  |  | 2 |  | 1 |  |  |
| 2 | 8 | 5 | 41 | Nursing, Dementia | Private | 46-66 | F=3, M=1 | 1 | 1 |  |  |  | 1 |  | 1 |
| 3 | 5 | 2 | 42 | Nursing, Dementia | Private | 31-45 | F=3, M=2 |  |  | 1 |  | 1 | 1 | 1 | 1 |
| 4 | 4 | 2 | 80 | Residential, Dementia | Private | 25-57 | F=3, M=2 |  |  | 1 |  |  | 3 | 1 |  |
| 5 | 10 | 1 | 40 | Residential, Dementia | Voluntary/Not-for-profit | 18-57 | F=6 |  |  | 4 |  |  | 2 |  |  |
| 6 | 10 | 1 | 35 | Nursing, Dementia | Voluntary/Not-for-profit | 26-60 | F=5 |  |  | 3 |  | 1 |  | 1 |  |
| 7 | 6 | 5 | 40 | Residential, Nursing & Dementia | Private | 29-60 | F= 5, M=2 |  |  | 2 |  |  |  | 5 |  |

AC= Activity co-ordinator, A= Administrator, CW= Care worker, DA= Domestic assistant, DM= Deputy Manager M= Manager, RN= Registered Nurse, SCW= Senior Care Worker

Footnotes

SIMD: Scottish Index of Multiple Deprivation: <https://simd.scot> (1 least deprived to 10 most deprived)
Urban – Rural Classification: https://www.gov.scot/publications/scottish-government-urban-rural-classification-2022/pages/overview/ (1 large urban area to 6 remote rural area)
