# Supplementary material for: Bereavement practices within older adult care homes in Scotland: a focus group study
Source: BMJ Open. 2026 Feb 23;16(2):e115592. doi: 10.1136/bmjopen-2025-115592 (PMC12931547; doi:10.1136/bmjopen-2025-115592)
Supplement: online supplemental file 4 [file bmjopen-16-2-s004.docx]

| **Structured Practices or Interventions to Support Healthy Bereavement and Grief** |
| --- |
| Personalised condolence cards sent to residents' families  Staff attendance at funerals  *Death Cafe*- style events for residents and staff to discuss death and dying  *To Absent Friends* events (https://www.toabsentfriends.org.uk/)  Physical memorialisation e.g. benches, trees, flower bouquets from funerals  Namaste Care [1]  Online Supportive Conversations and Reflection Sessions (OSCaRS) [2]  Friends and relatives invited to volunteer at the care home  Leaflets available on what to do when someone dies  Digital legacies shared directly with relatives from residents' time in the care home  Organisation counselling services available to staff and their relatives  Activity co-ordinators increasing support for residents who have lost a friend  Written instructions in Care Home Contract on how/when to clear/collect belongings |

## References

1. Salvi, S., et al., Implementing Namaste Care in nursing care homes for people with advanced dementia: a systematically constructed review with framework synthesis. BMC Geriatrics, 2025. 25(1): p. 17.

2. Hockley, J., et al., Practicalities of promoting practice-based learning in end of life care for care home staff: Lessons from “online” supportive conversations and reflection sessions. International Journal of Older People Nursing, 2024. 19(1): p. e12598.
