# Supplementary material for: Bereavement practices within older adult care homes in Scotland: a focus group study
Source: BMJ Open. 2026 Feb 23;16(2):e115592. doi: 10.1136/bmjopen-2025-115592 (PMC12931547; doi:10.1136/bmjopen-2025-115592)
Supplement: Supplementary file 5 [file bmjopen-2025-115592.reviewer_comments.pdf]

PEER REVIEW HISTORY

BMJ Open publishes all reviews undertaken for accepted manuscripts. Reviewers are asked to complete a checklist review form and are provided with free text boxes to elaborate on their assessment. These free text comments are reproduced below.

ARTICLE DETAILS

Title (Provisional)

Bereavement practices within older adult care homes in Scotland: a focus group study

Authors

Drummond, Maria; Burton, Jennifer; MacEachen, Doreen; Johnston, Bridget Margaret

---

VERSION 1 - REVIEW

**Reviewer**	**1**
**Name**	**White, Nicola**
**Affiliation**	**University College London, Marie Curie Palliative Care Research Department**
**Date**	**09-Jan-2026**
**COI**	**None**

Thank you for sending the manuscript titled: Bereavement practices within older adult care homes in Scotland: a focus group study.

This is a qualitative study exploring how care home staff experience bereavement and provide bereavement care. The findings are novel and really interesting and relevant to the readership of the BMJ Open. The methods are robust and justified. The results are clear and reported in a logical manner. The quotes are used appropriately. The authors have included reflexivity in the results which is important. The discussion is a balanced reflection on the findings with important implications.

Overall, I think this is a really well written paper.

**Reviewer**	**2**
**Name**	**Goh, Hongli Sam**
**Affiliation**	**National University of Singapore**
**Date**	**11-Jan-2026**

**COI**	**None**

Thank you for the opportunity to read and review this manuscript. This study offers valuable insights into an area less commonly researched. The data analysis is rich in textual descriptions and align with the themes presented by the authors. The discussion is also well-discussed. The authors also did well to include the COREQ checklist to ensure its study robustness.

Here are some comments for the authors to consider enhancing their manuscript:

• Date Error/Confusion: Abstract: "Data were collected in October–November 2024 using fictional bereavement…" vs Page 6: "Seven focus groups were conducted between August and December 2024" – where is there a difference?

• Study setting: Provide additional information about the EOL care practices in Scotland care homes (Page 5, Line 5 -13) for the international readers. References are needed here.

• Sampling criteria: not mentioned. Why is this so?

• Interview guide/questions – not provided despite being mentioned in Appendix 1 (page 6). While I understand that the interview questions or research questions might be omitted within some qualitative approaches to achieve reflexivity in findings, it is important to mention how the discussions were initiated or explored to guide the domain/themes generated. Additionally, the authors can consider including the fictitious scenarios to enhance their methodological transparency and study replicability across the 7 focus groups.

• Rigour – Page 6, Line 41 – 44. Can the authors consider including summary thematic table or analytic structure as part of its audit trail? This will allow the readers to understand how the authors generated the 3 themes (with supporting quotes). Additionally, while each theme seems to have subthemes, these are not obvious without any supporting diagrams or explicitly mentioned.

• Page 13 Line 48 – Page 14 Line 8: Please include how your study contributes to existing studies similar to the topic. It should be discussed as the authors mentioned limited existing literature in this area. The authors need to justify how their study add to the body of knowledge within the "Discussion" section.

• Consider including the implications of their studies under "Discussion"

• Appendix 2: requires heading – Page 20.

• Type: Page 10 Line 53 – "Namaste care": Shouldn't the "c" be capitalised?

VERSION 1 - AUTHOR RESPONSE

Reviewer 1 Comment	Response	
Thank you for sending the manuscript titled: Bereavement practices within older	We thank the reviewer for their careful reading of	

adult care homes in Scotland: a focus group study.<br>This is a qualitative study exploring how care home staff experience bereavement and provide bereavement care. The findings are novel and really interesting and relevant to the readership of the BMJ Open. The methods are robust and justified. The results are clear and reported in a logical manner. The quotes are used appropriately. The authors have included reflexivity in the results which is important. The discussion is a balanced reflection on the findings with important implications.	the manuscript and for their very positive and supportive feedback. We are pleased that the novelty, methodological rigour and clarity of reporting were recognised, and that the discussion was viewed as a balanced reflection of the findings. We appreciate the reviewer's indication that no revisions were required.	
**Reviewer 2 Comment**	**Response**	**Additional Text**
Date Error/Confusion: Abstract: "Data were collected in October–November 2024 using fictional bereavement…" vs Page 6: "Seven focus groups were conducted between August and December 2024" – where is there a difference?	Apologies, we have now made it clearer that recruitment was open between August 2024 and December 2024, but data were collected October-November 2024.	Recruitment was open between August and December 2024, with seven focus groups conducted between October to November of that period. – Page 6
Study setting: Provide additional information about the EOL care practices in Scotland care homes (Page 5, Line 5 -13) for the international readers. References are needed here.	Thank you to the reviewer for this suggestion, further context has been added with references.	Care home residents are a frail population who account for a significant proportion of deaths in older age groups in Scotland, reflecting the routine presence of dying and death within care home settings [7, 8, 24]. In Scotland, national policy frames palliative and end-of-life care as a priority centred on dignity, person-centred care and family support [25]. However, variation in practice and integration with wider services remains a continuing challenge. UK evidence highlights persistent difficulties in coordination between care homes and external services [26]. This disconnect becomes particularly visible when residents deteriorate. In these situations, care home staff regularly weigh complex risks and benefits under conditions of uncertainty, shaped by relationships and organisational pressures [27]. Nevertheless, most care homes report that they have the capacity to support and deliver palliative care.
Sampling criteria: not mentioned. Why is this so?	Thank you for this suggestion, clear inclusion criteria have been added.	Inclusion criteria: Eligible sites were care homes in Scotland registered with the Care

		Inspectorate that provided 24-hour care for older adults. Eligible participants were staff aged ≥17 years, employed in participating homes in any role with experience of resident death or bereavement practice, and able to provide informed consent. – Page 6
Interview guide/questions – not provided despite being mentioned in Appendix 1 (page 6). While I understand that the interview questions or research questions might be omitted within some qualitative approaches to achieve reflexivity in findings, it is important to mention how the discussions were initiated or explored to guide the domain/themes generated. Additionally, the authors can consider including the fictitious scenarios to enhance their methodological transparency and study replicability across the 7 focus groups.	Sorry for this oversight, the scenarios have now been added as an appendix 2 document.	
Rigour – Page 6, Line 41 – 44. Can the authors consider including summary thematic table or analytic structure as part of its audit trail? This will allow the readers to understand how the authors generated the 3 themes (with supporting quotes). Additionally, while each theme seems to have subthemes, these are not obvious without any supporting diagrams or explicitly mentioned.	We thank the reviewer for this thoughtful suggestion. During analysis, we worked with a more detailed analytic structure that included subthemes. However, in preparing the manuscript we deliberately streamlined the presentation to focus on the three overarching themes that corresponded to the Tapestry metaphor to maintain clarity and avoid over-fragmenting the findings within the journal word limits. We found that introducing additional subtheme labels added complexity without substantially enhancing interpretation, as the analytic distinctions are already embedded within the narrative description and supporting quotes.<br><br>To address the reviewer's request for transparency, we have added a summary thematic table (Table 2) that presents the	For analytic transparency and to provide an explicit audit trail, a summary thematic table (see Table 2) was developed linking codes, subthemes and overarching themes with illustrative quotes. – Page 7 and new table page 10

	analytic structure linking codes to the three themes with illustrative quotations. This provides an explicit audit trail while preserving the readability of the Results section.	
Page 13 Line 48 – Page 14 Line 8: Please include how your study contributes to existing studies similar to the topic. It should be discussed as the authors mentioned limited existing literature in this area. The authors need to justify how their study add to the body of knowledge within the “Discussion” section.	We thank the reviewer for emphasising the importance of clearly articulating the study’s contribution. The Discussion section is intended to situate our findings within the limited existing literature and to explain how this study adds to the current evidence base. Given journal word limits, we have not added a separate contribution paragraph; instead, we have revised the Discussion section to more explicitly signal how our findings extend previous work and address identified gaps in care home bereavement research. We believe this clarification strengthens the justification of the study’s contribution without substantially increasing manuscript length.	
Consider including the implications of their studies under “Discussion”	We thank the reviewer for this suggestion. The Discussion section already integrates practical and policy implications throughout the interpretation of findings. To make this clearer, we have added an explicit signposting sentence early in the Discussion to orient readers to the implications for workforce support, communication pathways and dementia-specific bereavement practice.	Together, these findings extend existing end-of-life literature by framing bereavement as a collective organisational practice rather than an individual emotional response. They have practical implications for how bereavement is recognised, supported and resourced within care homes, particularly in relation to workforce support, communication with external services and dementia-specific guidance.
Appendix 2: requires heading – Page 20.	Thank you, this has been added.	

Type: Page 10 Line 53 – “Namaste care”: Shouldn’t the “c” be capitalised?	Thank you for highlighting this continuity error. We have now capitalised it to be consistent with our Appendix 3 document.	Now on page 14
